# Editorial: Omics-driven crop improvement for stress tolerance

**DOI:** 10.3389/fpls.2023.1172228

**Published:** 2023-04-21

**Authors:** Weicong Qi, Jian Chen, Yi Han, Zhen Li, Xiaofeng Su, Freddy Kuok San Yeo

**Affiliations:** ^1^ Excellence and Innovation Center, Jiangsu Academy of Agricultural Sciences, Nanjing, China; ^2^ Key Laboratory of Saline-Alali Soil Improvement and Utilization (Coastal Saline-Alkali Lands), Ministry of Agriculture and Rural Affairs, Nanjing, China; ^3^ International Genome Center, Jiangsu University, Zhenjiang, China; ^4^ National Engineering Laboratory of Crop Stress Resistance Breeding, School of Life Sciences, Anhui Agricultural University, Hefei, China; ^5^ Vlaams Instituut voor Biotechnologie (VIB) Center for Plant Systems Biology, Department of Plant Biotechnology and Bioinformatics, Ghent University, Gent, Belgium; ^6^ Biotechnology Research Institute, Chinese Academy of Agricultural Sciences, Beijing, China; ^7^ Faculty of Resource Science and Technology Universiti Malaysia Sarawak (UNIMAS) Kota Samarahan, Sarawak, Malaysia

**Keywords:** omics, transcriptomics, genomics, metabolomics, plant breeding, abiotic/biotic stress

Crop losses due to biotic and abiotic stresses are significant worldwide issues. According to a report of the Food and Agriculture Organization of the United Nations (FAO), an estimated 20-40% of global crop production is lost every year due to pests and diseases alone, while other environmental factors, like drought, floods, high salinity level in soil, and extreme temperatures contribute to the losses becoming even more severe. Crop yield stability and healthy growth under biotic and abiotic stresses have always been a major challenge for the plant/agricultural researchers. Crop resilience is an important trait, and it involves essential phenotypes that plant breeding researchers are concerned with. For instance, fusarium-head-blight resistance is highly desirable for breeding new wheat varieties nowadays. Therefore, improving stress tolerance becomes a major research direction in modern crop sciences.

Currently, the thriving omics technologies have revolutionized plant sciences because they facilitate researchers to comprehensively analyze biological processes from multiple dimensions and draw an atlas with more detailed overview of genomic and phenotypic profiles in different environmental conditions. These technologies include genomics, transcriptomics, proteomics, metabolomics, and phenomics, among others. Genomics allows researchers to sequence the entire genome of plants, which can reveal important genetic information relevant to stress-tolerance, yield potential, etc. Transcriptomics enables the identification of gene expression variation in specific tissues or their response to environment, stress, and stimulation. While proteomics provides insights into the nucleotide sequences translation and modification in various biological processes. Metabolomics could be engaged to study the complex network of biochemical reactions, chemical signals, specific metabolites accumulation, and so on. Phenomics enable constantly monitoring plant development traits, for instance, plant height, leaf size, as well as physiological characters like photosynthesis efficiency, nutrient absorption, water consumption, etc.

Omics technologies provide new opportunities for plant breeding and food development, which is enormously vital for the modern world facing challenges such as climate change, global warming, and population expansion. This research topic has welcomed research/review articles describing the application of omics technologies in crop sciences, and a total of 20 studies were collected. Most of the contributions employed genomics, transcriptomics, metabolomics, and other omics technologies to study various stress factors like drought, salt stress, fungi infection, nutrient starvation, as well as hormone and nanomaterial treatments, etc. All the investigations were carried out on crops such as quinoa (Luo et al.), rice (Liang et al.), potato (Qin et al.), strawberry (Chen et al.), orange (Duan et al.), tea (Hu et al.; Chen et al.), cotton (Lu et al.), ornamental crop rose (Liu et al.), and so on.

Quinoa (*Chenopodium quinoa* willd.) is a novel crop that gained popularity worldwide in the last decades and has been widely employed in the infant and healthy food market. Luo et al. investigated the long noncoding RNA (LncRNA) in transcriptomic data of quinoa under salt stress.

Chen et al. analyzed LncRNA in ripening strawberry fruit treated with Abscisic acid (ABA). ABA is a well-known hormone associated with plant stress tolerance and seed or fruit maturation. In their study, transposon-derived LncRNAs and ABA-responsive LncRNAs were identified, and critical metabolomic pathways involved in the ripening process of strawberry fruit were predicted.

Liu et al. conducted research on the resistance of rose petal to powdery mildew, which is caused by *Podosphera pannosa*, and could be devastating in postharvest process of the cut flower. Transcriptomic analysis indicated that ethylene is playing an important role in the infection process, and ethylene inhibitor treatment could alleviate the symptom of inoculation.

Yan et al. investigated calcium nutrition nanoagent applying to tomato, of which the result demonstrated the nanomaterial could rescue the plant from mosaic virus disease.

The contribution of Xu et al. indicated a histone deacetylase inhibitor could enhance rice immunity to rice blast. Based on ChIP-seq, ChIP-qPCR and transcriptome data, the mechanism was probed in depth.

Lu et al. examined the hormone and signaling pathways involved in *AmCBF1* transcription factor coding gene overexpression-induced dwarfism in cotton. With RNA sequencing, they identified *PP2C* gene could be relevant with AmCBF1 and mediating plant architecture. Their further yeast one-hybrid and dual-luciferase results validate this hypothesis.

Overall, current Research Topic demonstrates a collection of scientific studies on various crop plants and provides robust examples highlighting the importance of omics technologies in understanding plant stress responses. These results shed new light and greatly advanced our understanding of plant molecular biology. Hopefully, these results and data presented in the topic, will serve as valuable reference and dataset for researchers working on crop breeding purposes, agronomic trait improvement, food quality elevation, and sustainable agriculture development.

## Author contributions

All authors listed have made a substantial, direct, and intellectual contribution to the work and approved it for publication.

